# Capacity and Adaptations of General Practice during an Influenza Pandemic

**DOI:** 10.1371/journal.pone.0069408

**Published:** 2013-07-18

**Authors:** Kristian A. Simonsen, Steinar Hunskaar, Hogne Sandvik, Guri Rortveit

**Affiliations:** 1 Research Group for General Practice. Department of Global Public Health and Primary Care, University of Bergen, Bergen, Norway; 2 Research Unit for General Practice, Uni Health, Uni Research, Bergen, Norway; 3 National Centre for Emergency Primary Health Care, Uni Health, Uni Research, Bergen, Norway; Centers for Disease Control and Prevention, United States of America

## Abstract

**Background:**

GPs play a major role in influenza epidemics, and most patients with influenza-like-illness (ILI) are treated in general practice or by primary care doctors on duty in out-of-hours services (OOH). Little is known about the surge capacity in primary care services during an influenza pandemic, and how the relationship between them changes.

**Aim:**

To investigate how general practice and OOH services were used by patients during the 2009 pandemic in Norway and the impact of the pandemic on primary care services in comparison to a normal influenza season.

**Materials:**

Data from electronic remuneration claims from all OOH doctors and regular GPs for 2009.

**Methods:**

We conducted a registry-based study of all ILI consultations in the 2009 pandemic with the 2008/09 influenza season (normal season) as baseline for comparison.

**Results:**

The majority (82.2%) of ILI consultations during the 2009 pandemic took place in general practice. The corresponding number in the 2008/09 season was 89.3%. Compared with general practice, the adjusted odds ratio for ILI with all other diagnoses as reference in OOH services was 1.23 (95% CI, 1.18, 1.27) for the 2008/2009 season and 1.87 (95% CI, 1.84, 1.91) for the pandemic influenza season. In total there was a 3.3-fold increase in ILI consultations during the pandemic compared to the 2008/09 season. A 5.5-fold increase of ILI consultations were observed in OOH services in comparison to the 2008/09 season. Children and young adults with ILI were the most frequent users of OOH services during influenza periods.

**Conclusions:**

The autumn pandemic wave resulted in a significantly increased demand on primary care services. However, GPs in primary care services in Norway showed the ability to increase capacity in a situation with increased patient demand.

## Introduction

Influenza epidemics occur almost every winter in the northern hemisphere [1], [2]. In April 2009 a new influenza virus characterized as a pandemic strain infected and spread rapidly in Mexico and USA [3]. The first confirmed case in Norway with influenza A(H1N1)pdm09 was reported in May that year, and Norway had two waves of influenza during the pandemic with the main wave in November 2009 [4], [5]. Norwegian health authorities have estimated that approximately 900,000 individuals out of a population of approximately 5 million were infected, and 32 laboratory confirmed deaths were reported [6].

The health authorities in Norway advised inhabitants with influenza symptoms to stay at home and allowed up to 7 days sick-leave without a sickness certificate in order to decrease influenza spread and pressure on the health care system [7]. Later on, oseltamivir was released at pharmacies as an over-the-counter-drug for influenza-like-illness (ILI). Despite these measures, a large number of ILI consultations took place in general practice or out-of-hours (OOH) services during the pandemic.

In many countries general practitioners (GPs) play a major role in influenza epidemics, and most patients with ILI are treated in general practice or by primary care doctors on duty in OOH services [8]–[11]. Accordingly, the extra workload of influenza patients puts pressure on the primary care service during epidemics, and this is added to normal activity [10]. General practices in Norway generally pre-book appointments and have a few available appointments for acute illnesses every day. In contrast, OOH services are organized with an empty schedule at the start of the shift. This organizational difference to tackle acute illnesses between day practice and OOH services may affect the ability to adapt to situations with high pressure. Little is known about how the surge capacity in these two primary care services, and how the relationship between them changes.

The first aim of this study was to investigate how and to what extent general practice and OOH services were used during the 2009 pandemic in Norway. The second aim was to investigate the impact of the pandemic on primary care services in comparison to a normal influenza season. The third aim was to investigate whether there were socio-demographical differences between patients with ILI treated in general practice and OOH services during a pandemic.

## Methods

### Ethics Statements

The project was approved by the Norwegian Social Science Data Services (project number 25159); stating that the project was based on registry data from Norwegian Health Economics Administration (HELFO). It was a pure registry study, and the selection will not be contacted. The data set that was delivered from HELFO was anonymous. This means that it was not possible to bring information back to individuals, either by name/social security number or reference of such information, or through sufficient background variables such as place of residence combined with diagnosis, gender, age etc. As personal data were not processed with electronic devices, and person registry containing sensitive personal information was not created, the project was not subject to notification requirements under the Personal Data Act § 31 and § 33.

### Data Source and Variables

In Norway primary care is organized by the municipalities [12]. In this study, we define primary care as general practice (service by GPs at day-time) and OOH services. The country has a registered list system administered by HELFO. There are more than 4,100 registered GPs with a total list population of approximately 5 million inhabitants (99.6% of the population) [13]. Emergency medical service is usually provided by the patient’s GP during office hours and by OOH services run by GPs on duty. In the largest cities the emergency service is also open at office-hours. In some of the larger cities, the 24-hour emergency service uses the coding system ICD-10 during day-time, although being part of the community health services. Activities at office hours from these services are not included in this material. The diagnosis system in primary care in Norway is based on International Classification of Primary Care (ICPC-2) coding system.

The Norwegian regular GP scheme is financed as fee-for-services. We examined data from electronic remuneration claims from all OOH doctors and regular GPs for 2009. Remuneration claims that were paper-based (3.4% of all contacts in 2006) are not included in the analyses [14]. The data file was delivered by HELFO and contained no person identifiable information. The following HELFO variables were used in this study: Patients’ age and gender, date and time of contact, type of contact (consultation at the office) and diagnosis according to ICPC-2. In addition, the centrality of the patient’s municipality was recorded. Centrality is defined as a municipality’s geographical location in relation to a centre where there are important central functions and is measured on a scale of 0 to 3 where 0 is the least and 3 is the most central.

### Case Definition and Definition of Influenza Periods

A clinical influenza case was defined as a consultation in which the ICPC-2 code “R80 Influenza/R80 Influenza-like illness” was used. All other diagnosis codes were grouped as “non-influenza”. A system of 201 sentinel GP practices is established in Norway by the Norwegian Notification System for Infectious Diseases, and they report the number of ILI weekly, from week 40 in autumn and to week 20 in spring [15]. In the pandemic season, this surveillance was extended to year-round reporting. The threshold for influenza season, as defined by Norwegian Institute of Public Health, is 1.4% ILI consultations per week. For the 2008/09 influenza season (an ordinary influenza season), this corresponds to week 1–9 2009, and for the pandemic influenza season this corresponds to week 30–51 2009. The 2008/09 influenza season was used as an ordinary influenza season because the level of ILI and the duration of the influenza period this season corresponded well to the average of ILI seasons 2006–2008, and the data for this period were readily available [16].

### Statistics

The data were analysed in IBM SPSS Statistics 19 with frequency analyses and cross-tabulations, as well as multivariate logistic regression analyses. In the frequency tables, age was dichotomized to the age groups 0–20 years and >20 years of age. Age and gender were considered as potential confounders and effect modifiers. Effect modification was tested by the Breslow–Day test for homogeneity between odds ratios (OR) after stratified analyses. Effect modification was statistically significant for age and gender on the association between the exposure variable (diagnosis) and the outcome variable (practice type) (data not shown). Confounding was evaluated by Mantel–Haenszel common odds ratios and logistic regression analyses. Multiple logistic regression analyses were performed to adjust for the confounders. We used multiple logistic regression analyses with practice type (general practice as reference category) as dependent variable and diagnosis (non-influenza as reference category) as explanatory variable. The multivariable analyses were adjusted for age, gender and centrality. Age was divided in three strata (0–20, 21–49 and 50 years and above) in the multivariable analyses. Significance was accepted at the 5% level (p < 0.05), and odds ratios were presented with 95% confidence interval (CI).

## Results

In 2009, there were 12,219,431 and 1,223,777 consultations in general practice and OOH services respectively; of these, 152,969 and 29,403 were consultations for ILI. In the pandemic season there was a 3.3-fold increase in total ILI consultations compared to the 2008/09 influenza season (3.0-fold increase of ILI in general practice and 5.5-fold increase of ILI in OOH services).

### Pandemic Influenza Season (Week 30–51 2009)

The total number of consultations for ILI during the pandemic is given in Table 1. Consultation for ILI peaked in week 44, with 14,087 consultations in general practice and 4,665 consultations in OOH services. The mean age of ILI patients was 29.4 years. There was a tendency for the youngest ILI patients to use OOH services more than their older counterparts (26.1% in age group≤20 years and 13.5% in age group>20 years). There were no gender differences in ILI visits to primary care services. There was a small geographical difference in the way patients with ILI visited primary care. In rural areas, 16.5% of ILI patients used OOH services, and in urban area 14.3% of ILI patients used OOH services.

**Table 1:** Consultations in general practice and out-of-hours (OOH) services during 2008/09 influenza season (week 1–9 2009) and pandemic influenza season (week 30–51 2009) by age, sex, centrality of the municipality and diagnosis (influenza vs. non-influenza).

		2008/09 influenza season				Pandemic influenza season			
		General practice		OOH services		General practice		OOH services	
	*Diagnosis* 1	No	%	No	%	No	%	No	%
*Consultations*	All	2,223,677	91.1	215,917	8.9	5,442,624	91.6	496,529	8.4
									
	ILI	34,441	89.3	4,116	10.7	104,168	82.2	22,632	17.8
	Other	2,189,236	91.2	211,801	8.8	5,338,456	91.8	473,897	8.2
*Age*									
0–20 years	ILI	5,153	79.8	1,301	20.2	32,343	73.9	11,412	26.1
	Other	360,622	81.2	83,619	18.8	756,571	81.7	169,642	18.3
>20 years	ILI	29,288	91.2	2,815	8.8	71,825	86.5	11,220	13.5
	Other	1,828,614	93.4	128,182	6.6	4,581,885	93.8	304,255	6.2
*Gender*									
Male	ILI	15,590	89.1	1,913	10.9	46,311	81.6	10,465	18.4
	Other	915,413	90.3	98,595	9.7	2,211,176	90.8	224,309	9.2
Female	ILI	18,851	89.5	2,203	10.5	57,857	82.6	12,167	17.4
	Other	1,273,823	91.8	113,206	8.2	3,127,280	92.6	249,588	7.4
*Centrality groups* 2									
Rural	ILI	12,444	90.0	1,385	10.0	35,907	83.5	7,008	16.5
	Other	937,658	93.0	70,283	7.0	2,314,286	93.3	166,352	6.7
Urban	ILI	21,359	91.7	1,930	8.3	66,012	85.7	11,008	14.3
	Other	1,168,140	93.0	88,272	7.0	2,858,137	93.4	200,806	6.6

1 ILI: Consultations with ICPC-2 code R80 influenza/influenza-like illness. Other: Non-influenza consultations, i.e. different ICPC-2 diagnoses.

2 The centrality is defined as a municipality’s geographical location in relation to a centre where there are important functions (central functions) and is measured on a scale of 0–3 where 0 is the least and 3 is the most central. Values are dichotomised to rural (0, 1 and 2) and urban (3).

### 2008/09 Influenza Season (Week 1–9 2009)

The total number of consultations for ILI in the 2008/09 influenza season is given in Table 1. The mean age of ILI patients was 36.5 years. There was a tendency for the youngest ILI patients to use OOH services more than their older counterparts (20.2% in age group≤20 years and 8.8% in the age >20 years). There were no gender differences among ILI patients. OOH services had 10.0% and 8.3% of ILI visits in rural and urban areas, respectively.

**Figure 1:**
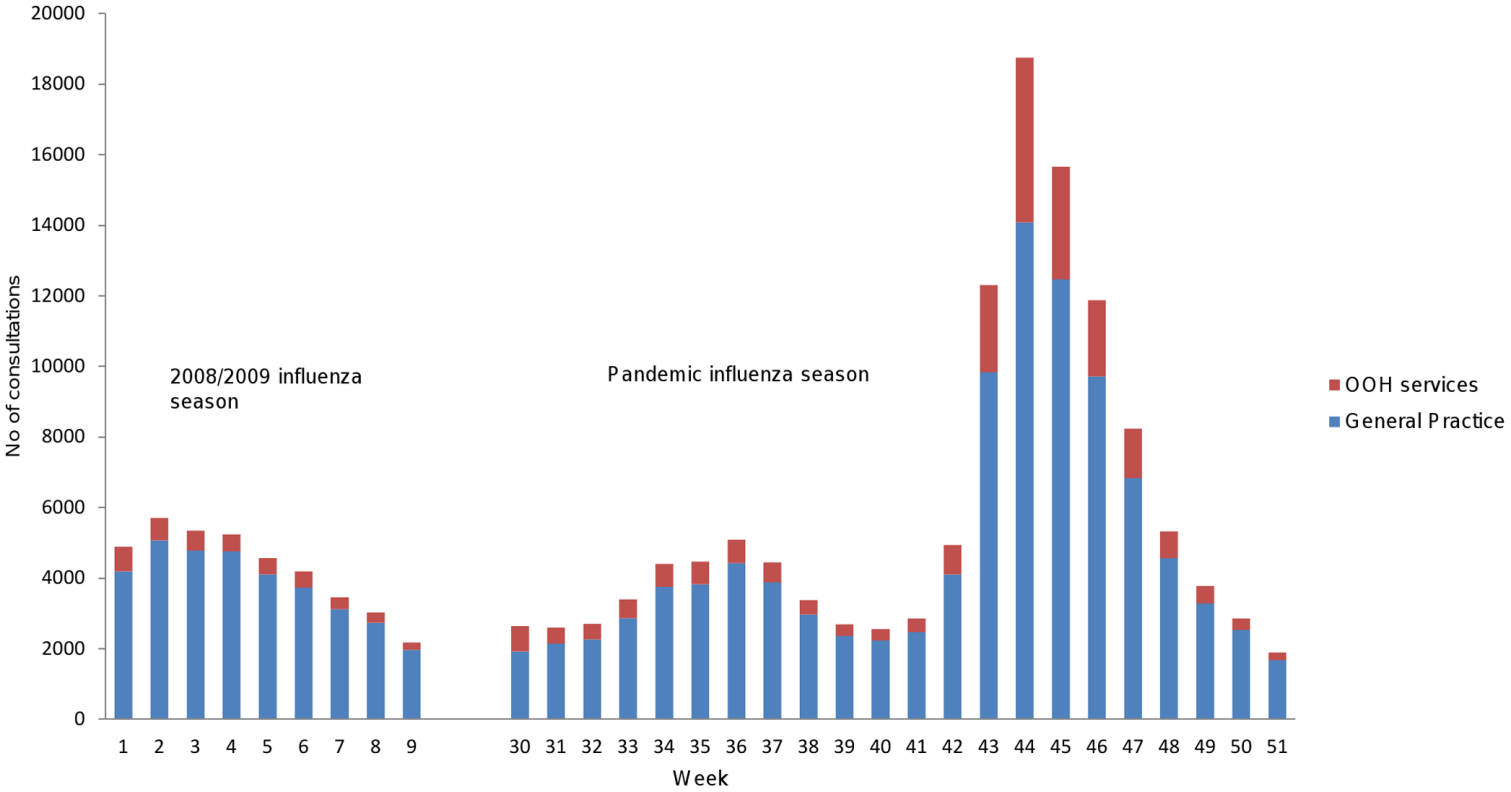
ILI consultations in general practice and out-of-hours services during 2008/09 influenza season (week 1–9 2009) and pandemic influenza season (week 30–51 2009).

**Figure 2:**
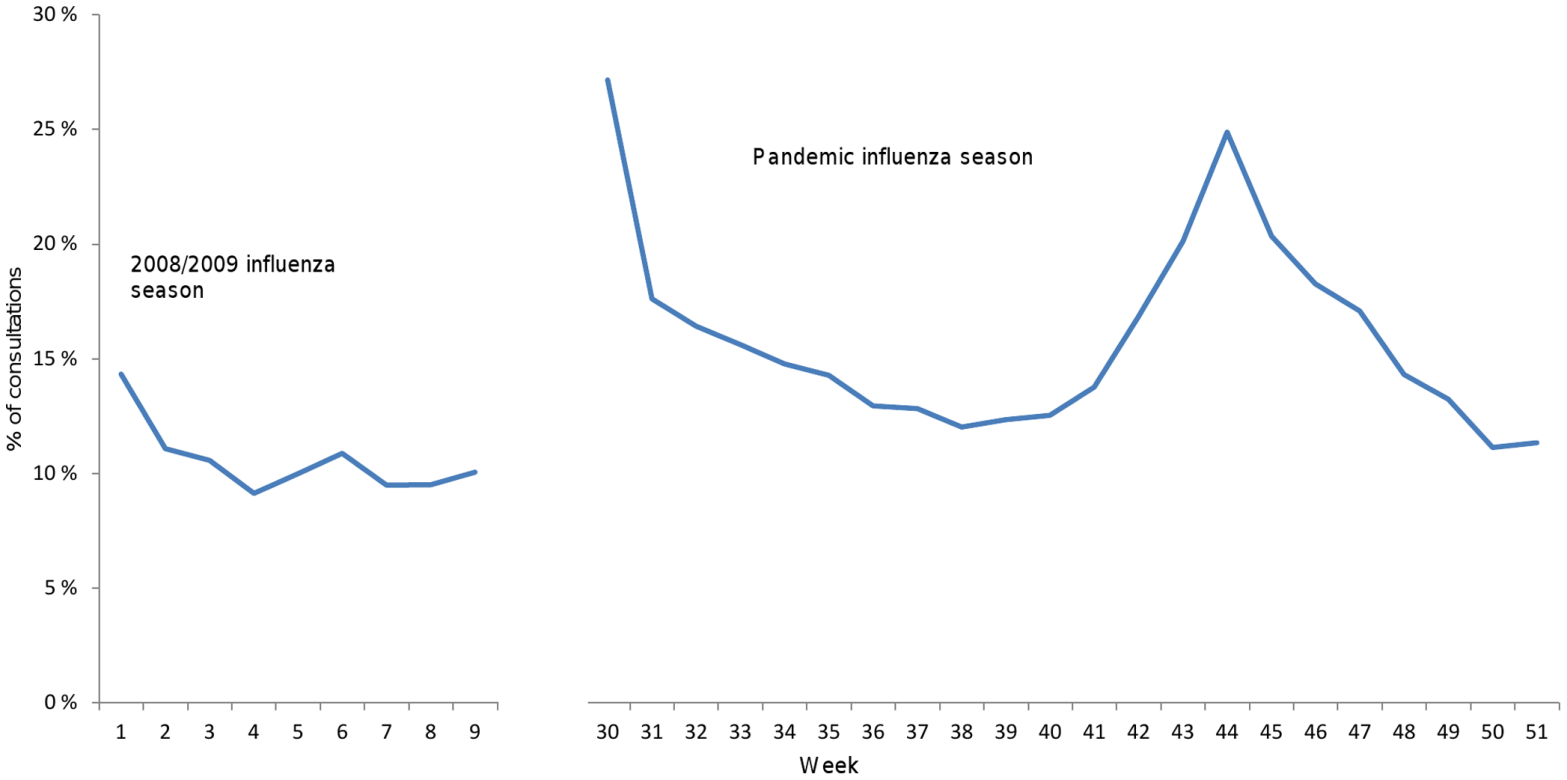
Proportion of ILI in out-of-hours services out of all ILI during 2008/09 influenza season (week 1–9 2009) and pandemic influenza (week 30–51 2009) season in Norway.

Figure 1 shows the total number of ILI consultations in both influenza periods. The pandemic influenza season started in week 30 and during the first 12 weeks of the pandemic, on average 15% of ILI consultations were conducted in OOH services, and the remaining in general practice. In week 42 there was a substantial increase in total number of ILI consultations and also a relative increase in consultations in OOH services. A two-fold increase in ILI visits was seen from week 42–43 in general practice and a four-fold increase in the OOH services in comparison to the weeks preceding the main wave. ILI consultations peaked in week 44, and at this point 25% of all ILI consultations were conducted in the OOH services. ILI consultations then subsided steadily over the next 7 weeks to end the pandemic influenza season in week 51. In the 2008/09 influenza season lasting nine weeks, on average 90% of all ILI visits took place in general practice, and the remaining in the OOH services. Figure 2 shows the proportion of ILI in OOH services out of all ILI during both influenza seasons.

Compared with general practice, the adjusted odds ratio for ILI with all other diagnoses as reference in OOH services was 1.23 (95% CI, 1.18, 1.27) for the 2008/2009 season and 1.87 (95% CI, 1.84, 1.91) for the pandemic influenza season (Table 2).Young age and living in rural areas were associated with higher OR for attending OOH services in comparison to general practice for both influenza seasons for any reason of encounter.

**Table 2:** Odds ratio (OR) for attending out-of-hours (OOH) services as compared with general practice during the 2008/09 influenza season (week 1–9 2009) and pandemic influenza period (week 30–51 2009).

	2008/09 influenza season				Pandemic influenza season			
Variable	Unadjusted OR	95% CI	Adjusted OR	95% CI	Unadjusted OR	95% CI	Adjusted OR	95% CI
*Diagnosis*								
Non-influenza	Ref.		Ref.		Ref.		Ref.	
Influenza	1.24	1.20, 1.28	1.23	1.18, 1.27	2.45	2.41, 2.48	1.87	1.84, 1.91
*Gender*								
Male	Ref.		Ref.		Ref.		Ref.	
Female	0.83	0.82, 0.83	0.85	0.85, 0.86	0.79	0.79, 0.80	0.82	0.82, 0.83
*Age*								
≥50 years	Ref.		Ref.		Ref.		Ref.	
21–49 years	1.60	1.59, 1.62	1.72	1.70, 1.74	1.77	1.76, 1.79	1.86	1.85, 1.88
0–20 years	4.23	4.18, 4.28	3.52	3.47, 3.57	4.56	4.53, 4.59	3.77	3.74, 3.80
*Centrality* 1								
3	Ref.		Ref.		Ref.		Ref.	
2	0.87	0.87, 0.90	0.92	0.90, 0.93	0.93	0.92, 0.94	0.97	0.96, 0.98
1	1.09	1.07, 1.11	1.12	1.10, 1.15	1.09	1.08, 1.10	1.14	1.12, 1.15
0	1.17	1.15, 1.18	1.24	1.22, 1.25	1.16	1.15, 1.18	1.26	1.25, 1.27

Multiple logistic regression analyses using practice type (general practice (ref.) and OOH services) as dependent variable and diagnosis, gender, age and centrality as explanatory variables.

1 The centrality is defined as a municipality’s geographical location in relation to a centre where there are important functions (central functions) and is measured on a scale of 0–3 where 0 is the least and 3 is the most central.

## Discussion

The majority of ILI consultations took place in general practice, both in the 2008/09 season and during the pandemic influenza season; however, there was a 5.5-fold increase of ILI consultations in the OOH services during the 2009 pandemic season in comparison to the 2008/09 season. Patients with ILI were younger during the 2009 pandemic compared to the previous influenza season. Younger ILI patients were more likely to use OOH services than general practice in both influenza seasons. We also found a geographical difference in the use of OOH services; rural areas in Norway had a slightly higher percentage of ILI patients in comparison to urban areas. There were no significant gender differences among ILI patients in this study with regard to visits to OOH services and general practice.

We used the 2008/09 season as a baseline or “normal” influenza season for comparison to the 2009 pandemic. Typically an influenza season lasts 8–10 weeks during winter months and our data shows that approximately 90% of all ILI consultations were handled in general practice at day-time, and the remaining were handled in the OOH services. A similar health utilization pattern was seen at the start of the pandemic influenza season, except in the very first part of the period when we had summer holiday in Norway and the capacity in general practice was reduced. However, as ILI visits in primary care were steadily increasing during the main autumn wave, a different type of adaption was seen. First the OOH services increased the capacity for ILI patients. Then, as the autumn wave emerged the same adaptation was seen in general practice. In week 44, the peak of the pandemic, 25% of all ILI patients were handled in the OOH services. However, the greatest number of encounters and hence largest capacity for ILI patients during the pandemic was found in general practice. This is logical, given that significantly more GPs are at work every day compared to OOH doctors. Additionally, the capacity for non-influenza consultations was increased in general practice during the autumn pandemic wave so that the total capacity for consultations increased. In the OOH services the total capacity was unchanged compared to pre-pandemic phase so that non-influenza consultations decreased to the benefit for influenza patients (data not shown). The increased workload on GPs during an influenza season is described before [10], [17], [18], but to our knowledge the interaction between general practice and OOH services during an influenza pandemic has not been studied previously. Out of 900,000 assumed infected individuals in Norway [6], our data implies that around 14% visited a primary care doctor. The real number is probably lower because some patients may have multiple encounters with the GP due to ILI. However, the large majority of the infected patients were not in need of a consultation with a GP during the pandemic. A study from OOH services in Norway reported that 75% of all encounters regarding influenza were managed by telephone consultation [19], and telephone consultations are more likely among young people and when disease is of minor severity [10]. Other reasons for the low GP consultation rate for influenza could be the introduction of the expanded use of sick-leave and the release of oseltamivir at pharmacies’ without prescription. From other studies it is supported that many cases were subclinical in nature [20], [21]. Successful mass vaccination could also have contributed to lower morbidity than first expected [4].

To our knowledge, this is the largest registry-based study on influenza conducted in primary care during the influenza pandemic in 2009. The study contains almost complete physician visit data from general practice and OOH services in Norway. However, our data have some limitations. The compensation claims are not designed for research purposes and contains no clinical data other than diagnosis. Another limitation is the risk of misclassification of other respiratory tract infections, which may have been under-diagnosed in the influenza seasons, whereas ILI may have been correspondingly over-diagnosed. The summer wave of “ILI” was mainly due to rhinovirus infection [22]. It is not possible to quantify the amount of misclassification in this study. However, to address the overall pressure on the primary care system during an epidemic and mechanism of adaptations in general practice and OOH services, it is of less importance to know the exact ILI incidence in the community. Misclassification may interfere with the analyses of socio-demographic variables so that the association between ILI and the use of health services is skewed. Misclassification of the disease may influence the associations between ILI and the use of health services. Misclassification of the disease (influenza) is a potential problem in primary care because the diagnostics relies on the interpretation of clinical features alone. At the best, GPs diagnose 60–70% of true influenza cases when the prevalence of influenza is high in the community [23].

In conclusion, the majority of ILI consultations during the 2009 pandemic took place in day-time general practice. Children and young adults with ILI were the most frequent users of OOH services during influenza periods. The autumn pandemic wave resulted in a significantly demand on primary care services. However, GPs in primary care services in Norway have the ability to increase capacity at situations with increased patient encounters.
